# High frequency of phylogenetically diverse reductive dehalogenase-homologous genes in deep subseafloor sedimentary metagenomes

**DOI:** 10.3389/fmicb.2014.00080

**Published:** 2014-03-03

**Authors:** Mikihiko Kawai, Taiki Futagami, Atsushi Toyoda, Yoshihiro Takaki, Shinro Nishi, Sayaka Hori, Wataru Arai, Taishi Tsubouchi, Yuki Morono, Ikuo Uchiyama, Takehiko Ito, Asao Fujiyama, Fumio Inagaki, Hideto Takami

**Affiliations:** ^1^Geomicrobiology Group, Kochi Institute for Core Sample Research, Japan Agency for Marine-Earth Science and Technology (JAMSTEC)Nankoku, Japan; ^2^Microbial Genome Research Group, Institute of Biogeosciences, Japan Agency for Marine-Earth Science and Technology (JAMSTEC)Yokosuka, Japan; ^3^Department of Bioscience and Biotechnology, Kyushu UniversityFukuoka, Japan; ^4^Comparative Genomics Laboratory, Center for Information Biology, National Institute of GeneticsMishima, Japan; ^5^Geobio-Engineering and Technology Group, Submarine Resources Research Project, Japan Agency for Marine-Earth Science and Technology (JAMSTEC)Nankoku, Japan; ^6^National Institute for Basic Biology, National Institutes of Natural SciencesOkazaki, Japan; ^7^Department of Basic Biology, School of Life Science, The Graduate University for Advanced StudiesOkazaki, Japan; ^8^Department of Biological Sciences, Tokyo Institute of TechnologyYokohama, Japan

**Keywords:** deep biosphere, dehalogenation, metagenome, *rdhA*, sedimentary habitat

## Abstract

Marine subsurface sediments on the Pacific margin harbor diverse microbial communities even at depths of several hundreds meters below the seafloor (mbsf) or more. Previous PCR-based molecular analysis showed the presence of diverse reductive dehalogenase gene (*rdhA*) homologs in marine subsurface sediment, suggesting that anaerobic respiration of organohalides is one of the possible energy-yielding pathways in the organic-rich sedimentary habitat. However, primer-independent molecular characterization of *rdhA* has remained to be demonstrated. Here, we studied the diversity and frequency of *rdhA* homologs by metagenomic analysis of five different depth horizons (0.8, 5.1, 18.6, 48.5, and 107.0 mbsf) at Site C9001 off the Shimokita Peninsula of Japan. From all metagenomic pools, remarkably diverse *rdhA*-homologous sequences, some of which are affiliated with novel clusters, were observed with high frequency. As a comparison, we also examined frequency of dissimilatory sulfite reductase genes (*dsrAB*), key functional genes for microbial sulfate reduction. The *dsrAB* were also widely observed in the metagenomic pools whereas the frequency of *dsrAB genes* was generally smaller than that of *rdhA*-homologous genes. The phylogenetic composition of *rdhA*-homologous genes was similar among the five depth horizons. Our metagenomic data revealed that subseafloor *rdhA* homologs are more diverse than previously identified from PCR-based molecular studies. Spatial distribution of similar *rdhA* homologs across wide depositional ages indicates that the heterotrophic metabolic processes mediated by the genes can be ecologically important, functioning in the organic-rich subseafloor sedimentary biosphere.

## Introduction

Previous microbiological studies conducted in conjunction with scientific ocean drilling have demonstrated remarkably high microbial cell abundance in deep and old organic carbon-rich marine subsurface sediments in the margins of the Pacific Ocean (Parkes et al., 1994, 2000; Lipp et al., 2008). The biomass in the open Pacific Ocean sediments, where primary photosynthetic production in the overlying water column is very small (e.g., South Pacific Gyre), is generally several orders of magnitude lower than that in the marginal sedimentary areas (D'Hondt et al., 2009). The current estimate of the global subseafloor microbial biomass is 2.9 × 10^29^ cells, comprising ca. 0.6% of the total carbon in living biomass on our planet (Kallmeyer et al., 2012). These facts mean that microbial communities in organic carbon-rich sediments in the margins of the Pacific Ocean contribute significantly to the global subseafloor microbial biomass.

In general, microbial activity in deep marine sediments, regardless of location, is extraordinarily low because of the limitation of bio-available electron donors and acceptors (D'Hondt et al., 2004; Røy et al., 2012; Hoehler and Jørgensen, 2013). However, there are metabolically active and/or alive microbial populations in deep marine sediments. Using nano-scale secondary ion-mass spectrometry (NanoSIMS), it has been demonstrated that a large fraction of deep subseafloor microbial components are physiologically alive, i.e., incorporate stable isotope-labeled carbon and nitrogen substrates (Morono et al., 2011). Successful extraction and sequencing of RNA and visualization of RNA by fluorescent *in situ* hybridization have also indicated the presence of metabolically active microbial components in deep marine sediments (Schippers et al., 2005; Sørensen and Teske, 2006; Mills et al., 2012; Orsi et al., 2013b).

In the deep subseafloor sedimentary biosphere, microbial communities comprise phylogenetically diverse bacteria and archaea (Inagaki et al., 2003, 2006; Teske, 2006; Fry et al., 2008), as well as eukaryotes (Orsi et al., 2013a) and viruses (Engelhardt et al., 2013; Yanagawa et al., 2013). Since the microbial constituents found in deep marine sediments are phylogenetically distinct from known isolates, their metabolic and physiological functions also remain largely unknown. In fact, the metagenomic analysis of the Peru Margin sediments showed that a large fraction (86–94%) of the sequences did not code any homologs of known protein-coding genes (Biddle et al., 2008), suggesting that microbial communities in marine subsurface are functionally and evolutionarily quite distinct from other microbial ecosystems of the Earth's surface biosphere (Biddle et al., 2006, 2011). A genomic study of single cells isolated from marine subsurface sediments has revealed extracellular protein-degrading enzymes, suggesting that previously unidentified metabolic functions related to protein degradation and recycling are present in the uncultured but predominant archaeal constituents in the marine subsurface sediments, such as members of the Miscellaneous Crenarchaeotic Group (Lloyd et al., 2013). In addition, stable isotope probing of the benthic microbial community showed that archaeal membrane lipids are recycled for new biomass production without energy-consuming lipid synthesis steps (Takano et al., 2010). These observations consistently suggest that deep subseafloor microbial constituents have unique metabolic and physiological functions that make them well suited for long-term survival under energy-limited conditions (Hoehler and Jørgensen, 2013).

One of the possible electron-acceptor systems in anoxic marine sediments is an organohalide respiration pathway (Bossert et al., 2003). Previous microbiological studies of various terrestrial environments and isolates demonstrated that the members of the genus *Dehalococcoides* are known to utilize organohalides as the sole terminal electron accepter by means of reductive dehalogenases (Löffler et al., 2003), in which reductive dehalogenase genes (*rdhA*) are key functional genes for organohalide respiration pathways (Bossert et al., 2003). In marine sedimentary habitats, culture-independent molecular ecological surveys of 16S rRNA genes showed that members of the phylum *Chloroflexi*, including *Dehalococcoides*-relatives, are the predominant bacterial components (e.g., Biddle et al., 2006; Inagaki et al., 2006; adrian, 2009). In addition, dehalogenation activities were observed in marine sediments (Häggblom et al., 2003), e.g., reductive dehalogenation of 2,4,6-tribromophenol was detected in sediment such as at 4.7 m below the seafloor in the Nankai Trough and somewhere else (Futagami et al., 2009, 2013). PCR-based analysis using a conventional primer set (i.e., RRF2 and B1R; Krajmalnik-Brown et al., 2004) revealed the widespread presence of phylogenetically diverse *rdhA*-homologous genes. However, because of potential bias caused by primer sequence mismatches (Teske and Sørensen, 2008; Hoshino and Inagaki, 2013), it is assumed that there are as-yet-unexplored functional genes related to the dehalogenation pathway in marine subsurface sediments.

In this study, we focused on the diversity, frequency and distribution of *rdhA*-homologs in organic carbon-rich marine subsurface sediments off the Shimokita Peninsula of Japan. The samples used in this study were obtained during the *Chikyu* Shakedown Expedition CK06-06 in 2006 (Aoike, 2007), in which relatively high cell numbers (>10^7^ cells/cm^3^) were observed using an image-based cell count technique (Morono et al., 2009).

To obtain a comprehensive molecular overview of *rdhA*-homologous genes in marine subsurface sediments, we performed metagenomic analysis of sediment core samples at five depths to 107 m below the seafloor. This study allowed us to quantitatively assess the metagenomic pools through primer-independent phylogenetic analysis of *rdhA*-homologous genes, extending our current knowledge of the functional and evolutional contexts of deep subseafloor microbial communities.

## Materials and methods

### Sample collection

Sediment samples used in this study were collected from Site C9001 Hole C (41° 10.6380′ N, 142° 12.081′ E), which is approximately 80 km from the coast of Shimokita Peninsula, northeastern Japan, during the *Chikyu* Shakedown Expedition CK06-06 in 2006 (Aoike, 2007). This site is at the same location of the Integrated Ocean Drilling Program (IODP) Expedition 337 hole designated as IODP Site C0020 Hole A (Inagaki et al., 2012). The water depth at Site C9001 is 1180.5 m. The sample depths examined in this metagenomic study are 0.8 (Core 1H-1), 5.1 (Core 1H-4), 18.6 (Core 3H-2), 48.5 (Core 6H-3), and 107.0 (Core 12H-4) mbsf. Environmental parameters such as pore-water chemistry and total organic carbon have been measured (Aoike, 2007; Tomaru et al., 2009). After core recovery onboard the vessel, 20-cm-long whole round scores were immediately sampled, and then the innermost part of the core was sub-sampled using an autoclaved tip-cut-syringe. The samples were placed in a −80°C freezer until DNA extraction.

### DNA extraction

DNA was extracted with an ISOIL for Bead Beating kit (Nippon Gene, Tokyo, Japan) according to manufacturer protocol, except for an additional treatment with lytic enzyme (Morita et al., 2007). Briefly, 5 g of sediment sample was suspended in 9 mL of five-fold diluted lytic buffer solution and vortexed vigorously. The suspension was transferred into a new 50 mL Falcon tube containing zirconia/silica beads and then shaken with a ShakeMaster Auto (ver. 2.0, Bio Medical Science Inc., Tokyo, Japan) for 5 min. After adding 0.5 mL of lysozyme solution (2.0 mg/ml) to the tube, the suspension was incubated for 1 h at 37°C with gentle shaking and further incubated for 1 h at 55°C after the addition of 0.5 mL of proteinase K solution (20 mg/ml) and 0.6 ml of lytic buffer 20S. The suspension treated by bead-beating and lytic enzymes was centrifuged at 6500 × g for 15 min at 4°C. DNA in the supernatant was purified twice according to manufacturer instructions. The DNA was further purified using a MagExtractor Plant Genome kit (Toyobo, Osaka, Japan). The concentration of the extracted DNA was measured using a Qubit fluorometer with a Quant-iT dsDNA HS Assay kit (Life Technologies, Carlsbad, CA, USA).

### Whole genome amplification

To increase amount of high-molecular DNA to construct shotgun genome libraries from marine subsurface sediments, it was found to be necessary that all DNA samples except for Core 1H-1 (0.8 mbsf) were amplified with multiple displacement amplification (MDA) using a GenomiPhi DNA Amplification kit (ver. 2, GE Healthcare, Uppsala, Sweden) according to manufacturer instructions (Lipp et al., 2008). To decrease potential amplification bias, we conducted multiple reactions (10–60 reactions per sample) with reaction time as short as 1.5 h, then the product was pooled, and used for sequencing analysis. The incubation was performed at 30°C after denaturing DNA at 95°C for 3 min. The Phi29 DNA polymerase and exonuclease were then inactivated by incubation at 65°C for 10 min.

### DNA sequencing, assembly, and gene prediction

Shotgun genomic libraries were constructed from extracted DNA that was randomly sheared (2–3 kb in length) using a HydroShear (Digilab Inc., Marlborough, MA, USA) and the pCR-BluntII-TOPO vector (Life Technologies). Approximately 10 μg of DNA was used to produce ca. 40,000 shotgun clones from each sediment sample. Template DNA for sequencing was prepared by PCR amplification of the insert DNA using an ExTaq kit (TaKaRa Bio Inc., Otsu, Japan) and a GeneAmp PCR System 9700 (Life Technologies). Sequencing was carried out from both ends of the cloned fragment using a BigDye v3.1 kit on ABI3730 (Life Technologies) and DeNOVA (Shimadzu Co., Kyoto, Japan) DNA sequencers.

The shotgun reads without 16S rRNA genes were filtered for low quality residues, so that the longest subsequence of a read is kept where Phred quality score is 20 or more for 80% in 20 bp window with sliding window size of 20 bp. The trimmed reads of each metagenomic sample were assembled to generate a consensus sequence for a contig based on an alignment of reads in the contig using the PCAP program (Huang et al., 2003) with default parameters.

The MetaGeneAnnotator program (Noguchi et al., 2008) was employed to predict potential protein-coding sequences (CDS ≥ 20 amino acids) from the assembled sequences. Prior to gene prediction, low-quality sequences (Phred quality score <15) were masked by “X”s. The results were described below and summarized in Table S1.

### Orthologous gene analysis

Assignment of metagenomic genes to orthologous gene groups was carried out using a pre-calculated ortholog table constructed from genomic sequences available from databases in the National Center for Biotechnology Information (NCBI; http://www.ncbi.nlm.nih.gov/). The table was constructed from 1806 available genome sequences without redundant representation of species (Table S2) using the DomClust program (Uchiyama, 2006). The metagenomic genes from the sediments were then assigned to the ortholog table using the MergeTree program in the RECOG package (http://mbgd.nibb.ac.jp/RECOG/), which is based on the clustering tree of each orthologous group in the table. Distance rather than score was used to construct the ortholog table and to merge the metagenomic genes into the table in order to ensure proper evaluation of evolutionary distance (Kawai et al., 2011). Distance values were calculated from identity values by a BLASTP (Altschul et al., 1997) search using Kimura's correction formula for protein sequence distances (Kimura, 1983). Homology pairs considered for calculation were unidirectional best hits with *E* ≤ 0.001 and score ≥60.0. Parameters used for the DomClust and MergeTree programs were as follows: -d (use distance as a measure of relatedness), −Ohorizweight = 0.0 (skip estimation of horizontal transfer), −C80.0 (cutoff score for domain split), −V0.6 (alignment coverage for domain split), −n1 (minimum number of organisms in clusters to be output), −ne1 (minimum number of entries in clusters to be output), −p0.5 (ratio of phylogenetic pattern overlap for tree cutting), −HO (both homology clustering, i.e., skip the tree cutting, and ortholog clustering), −ai0.95 (member overlap for absorbing adjacent small clusters), and −ao0.8 (member overlap for merging adjacent clusters).

### Calculation of average coverage ratio

To normalize the differences in the total number of sequences in each horizon, an “average coverage ratio” for each gene was defined as the ratio of the average coverage value of the gene to the average of average coverage values of 35 universally conserved single-copy genes.

Nucleotide sequence coverage of a gene in a metagenomic sequence (“average coverage” value in short) was defined as the average number of sequence reads mapped to each nucleotide position of the gene and the value is calculated as the total number of nucleotides that overlapped the gene divided by the length of the gene. The expected length of a gene in metagenomes is taken as the average length of the orthologous genes identified in the reference genomes based on the fact that metagenomic genes are largely truncated at the ends of sequence reads. Average coverage was based on clones rather than on individual reads; two reads from the same clone were counted as one if they overlapped with each other on a single contig.

Average coverage value is dependent on the sequencing depth of a metagenomic sample (i.e., the total amount of metagenomic sequences determined). Hence, the different sequencing depth among different samples (in our case, five different depths) must be normalized to compare the amount of genes among different metagenomes. To normalize the different sequencing depths among samples, we used average of the average coverage of universally conserved single-copy genes, which are conserved in almost all of the archaeal and bacterial genomes, to represent the estimated sequencing depth defined by the number of estimated genome equivalent. The 35 universally conserved single-copy genes used in this study (Table S3) were selected from the ortholog table based on the following criteria: (i) universally conserved among archaeal genomes, i.e., present in 95% of 110 complete archaeal genomes among 122 archaeal genomes used in this study which includes 12 incomplete genomes (Table S2), (ii) universally conserved among bacterial genomes, i.e., present in 95% of 1055 complete bacterial genomes among 1657 bacterial genomes used in this study, and (iii) present as a single-copy gene in most of the genomes, i.e., the average copy number among complete archaeal and bacterial genomes is less than 1.2 for both (Table S3). The average coverage ratio of the universally conserved single-copy genes for the five metagenomes was generally around one (Table S3).

Thus, the calculated average coverage ratio indicates the number of copies of a gene per a genome equivalent in the metagenomic sequences.

Metagenomic sequences from the Peru margin sediment samples (Biddle et al., 2008) [GenBank:SRR001322-SRR001326] were analyzed similarly as those of the present study. Low quality reads and duplicated reads were eliminated using PRINSEQ (Schmieder and Edwards, 2011). Comparison of reads and the reference genomes was carried out using TBLASTN (Altschul et al., 1997). Phylogenetic assignment of each read was based on the top hit among the *rdhA* genes detailed in the present study.

### Statistical analysis

Statistical tests under hypergeometric distribution were conducted with Benjamini and Hochberg multi test correction to extract orthologous groups, in which the number of metagenomic genes assigned to the orthologous group was overrepresented compared to the number of reference genomes assigned to the group. In the test, the probability of deviation of the sample (the number of genes of the metagenomes) from the control (the number of genes of the reference genomes) was calculated based on the hypergeometric distribution, which is shown to be equivalent to the Fisher exact test (Rivals et al., 2007). If we randomly select *k* genes, where *k* equals the number of the metagenomic genes, from the all genes of the reference genomes and the metagenomic pools that are labeled as orthologous group X (*m*) or non-X (*n*), the distribution tells us the probability of getting *q* or more genes labeled as X. The sum of the average coverage of each gene (or that of a domain of the gene) of an orthologous group was rounded down to the nearest integer and used as the number of the metagenomic sample for the statistical test, while the number of genes assigned to the orthologous group was used for the reference genomes. Adjustment of *p*-values for multiple comparisons (multi-test correction) was performed using the Benjamini and Hochberg controlling procedure of false discovery rate (FDR) (Benjamini and Hochberg, 1995). All statistical analyses were conducted using the R statistical package (R Core Team, 2013).

### Taxonomic distribution

Ribosomal rRNA genes were searched for SILVA ribosomal RNA gene database (version 111) (Quast et al., 2013) using BLASTN (Altschul et al., 1997) with *E* ≤ 0.001 and -dust no. Count of fragments was based on clones rather than on individual reads; two reads from the same clone were counted as one if they overlapped with each other. If a region of a sequence matched both large subunit ribosomal rRNA and small subunit ribosomal rRNA genes, higher score hit was used. An rRNA gene fragment was identified when it had equal or more than 100 bp homology with an rRNA gene. In addition, if the matched region is internal part of a sequence, the whole part of the hit should match. This case is a match between a query and a hit shorter than the query (e.g., archaeal PCR clones in the database can be less than approximately 1 kb). Taxonomy information was assigned to each 16S rRNA gene fragment using the mother program (Schloss et al., 2009). Coverage value was calculated as the ratio of the total length of 16S rRNA gene fragments to the length of the top hit 16S rRNA gene in the database.

### Phylogenetic analysis

A phylogenetic tree was constructed with the putative *rdhA* genes in the metagenomes and the reference genomes, as well as PCR products of the *rdhA* genes detected in subseafloor sediments (Futagami et al., 2009) and several functionally characterized reductive dehalogenase genes (Table S4). The *rdhA*-homologous sequences in the metagenomes were defined by two steps; (1) choosing orthologous groups of *rdhA* genes, and (2) refinement based on phylogenetic tree, as described below. Included orthologous groups were those of which half or more of the genes in the cluster have the RdhA domain [TIGR02486 model in TIGRFAMs database (Haft et al., 2003)]. Identification of RdhA domain (TIGR02486) was conducted using the Hmmer3 program (Eddy, 2011) against the TIGRFAMs (Haft et al., 2003) using the noise cutoff bit score thresholds (–cut_nc). Amino acid sequences of these gene products were aligned using the einsi command of the MAFFT program (Katoh et al., 2005). To construct phylogenetic trees from the protein alignment, because the *rdhA* homologous fragments from the metagenomes were not all overlapping each other, we used the FastTree program (Price et al., 2010). This program can generate a reasonable topology of an approximately-maximum-likelihood phylogenetic tree using an alignment with some of the sequences do not overlap each other. The default parameters of the program were used as follows: Jones-Taylor-Thornton model of amino acid evolution, a single rate for each site (the “CAT” approximation) to account for the varying rates of evolution across sites, and support values with the Shimodaira-Hasegawa test to estimate the reliability of each split in the tree (Price et al., 2010). Specifically, the FastTree uses the SH test to compare (A,B), (C,D) to alternate topologies (A,C), (B,D) or (A,D), (B,C), given a topology (A,B), (C,D), where A, B, C, D may be subtrees rather than leaves. It has been recommended that the selection threshold for SH value should be in the 0.8–0.9 range (Guindon et al., 2010). The result showed that SH 0.85 as a threshold of branch selection have similar power as bootstrap (BP) 0.75 in the sense that about 85% of correct branches are selected. The results of simulated data sets also validated the common practice to use “a rule of thumb,” whereby sufficient evidence is indicated by BP value above 0.7–0.8, discarding most of the incorrect branches (for details, see Guindon et al., 2010). At the end, SH values 0.8 and 0.9 were used in this study to indicate the support value of each split. A second phylogenetic tree was constructed after eliminating the genes from subtrees for which the RdhA domain (TIGR02486) was not detected in any of the genes. Orthologous groups that included *dsrA* and *dsrB* genes of *Archaeoglobus fulgidus* (Dahl et al., 1993) were defined as *dsrA* and *dsrB*, respectively.

### Protein domain search

Identification of the protein domain was conducted using the Hmmer3 program (Eddy, 2011) against the TIGRFAMs (Haft et al., 2003) and Pfam (Finn et al., 2010) databases using the noise cutoff bit score thresholds (–cut_nc). Protein families in TIGRFAMs are intended to detect orthologous proteins to provide information with the maximum utility for the annotation process (Haft et al., 2003). On the other hand, protein families in Pfam typically achieve broad coverage across distant homologs but end at the boundaries of conserved structural domains (Finn et al., 2010). Hence, we complementarily used these two well-maintained protein family databases.

### Genomic context analysis

*rdhA* genes characterized to date sometimes accompany an short *rdhB*–homologous gene, which encodes a hydrophobic protein (Smidt and de Vos, 2004). Gene sequences within 2 kb of the *rdhA*-homologous genes were searched for putative *rdhB* genes. Orthologous groups defined as putative *rdhB* in this study were those of which genes have predicted amino acid sequences shorter than 150 amino acids and two to three transmembrane regions. Initial screening with amino acid sequences shorter than 200 amino acids revealed that homologous in the ortholog table of the previously described *rdhB* genes were below 150 amino acids in length [Table S5; the previously described *rdhB* genes are as follows: *pceB.Smu* from *Sulfurospirillum multivorans* (Neumann et al., 1998), *cprB.Dde* from *Desulfitobacterium dehalogenans* (van de Pas et al., 1999; Smidt et al., 2000), *vcrB.Dba* from *Dehalococcoidia* bacterium (Müller et al., 2004), *pceB.Dha* from *Desulfitobacterium hafniense* (Futagami et al., 2006), *dcaB.Ddi* from *Desulfitobacterium dichloroeliminans* (Marzorati et al., 2007)]. Homologs of these *rdhB* genes were also listed, as some of flanking genes of putative *rdhA* genes derived from the metagenomes were truncated at the end of a nucleotide sequence and unable to check the exact length and the number of transmembrane regions. The transmembrane domains were predicted using the TMHMM Server 2.0 program (Krogh et al., 2001). TMHMM is based on a hidden Markov model (HMM) approach. HMM is well suited for prediction of transmembrane helices because it can incorporate hydrophobicity, charge bias, helix lengths, and grammatical constraints into one model.

### Accession numbers

The nucleotide sequence data have been submitted to the DDBJ database under the following accession numbers: DDBJ:BARS01000001-DDBJ:BARS01060478 for 1H-1, DDBJ:BART01000001-DDBJ:BART01044241 for 1H-4, DDBJ:BARU01000001-DDBJ:BARU01051978 for 3H-2, DDBJ:BARV01000001-DDBJ:BARV01048200 for 6H-3, and DDBJ:BARW01000001-DDBJ:BARW01045500 for 12H-4.

## Results

### DNA extraction, sequencing, and gene prediction

The samples examined in this study were obtained from 0.8 (Core 1H-1), 5.1 (Core 1H-4), 18.6 (Core 3H-2), 48.5 (Core 6H-3), and 107.0 (Core 12H-4) meters below the seafloor (mbsf) at Site C9001 Hole C, off Shimokita Peninsula of Japan, during the drilling vessel *Chikyu* Shakedown Expedition CK06-06 in 2006 (Aoike, 2007). The amount of DNA extracted from samples at each depth was as follows: 0.42 μg/g (0.8 mbsf), 0.24 μg/g (5.1 mbsf), 0.18 μg/g (18.6 mbsf), 0.05 μg/g (48.5 mbsf), and 0.03 μg/g (107.0 mbsf). The depth profile of cell numbers reported earlier (in the order of 10^8^–10^9^ cells/cm^3^ at a few mbsf to 10^7^ cells/cm^3^ at 100 mbsf) (Morono et al., 2009) is consistent to the decreasing trend of the extracted DNA amount. The paired-end sequences of ca. 40,000 shotgun clones from each horizon were determined. The length of sequence reads ranged from 650 to 800 bp. The sum of sequence length of 76,000–77,000 reads was ca. 50 Mbp per each sample. The ratio of the total sequence length after assembly to that before assembly was 84 to 98% (Table S1). N50 of contigs was 1.5 to 1.7 kb (Table S1), suggesting that most contigs comprised two reads. Consensus sequences of the contigs were used in further analyses. The number of predicted genes in each sample was 86,362 (0.8 mbsf), 67,494 (5.1 mbsf), 73,184 (18.6 mbsf), 67,201 (48.5 mbsf), and 63,711 (107.0 mbsf) (Table S1). Sum of the nucleotide length of assembled nucleotide sequences that did not encode any genes homologous to those of reference genomes was less than 25% of the total assembled nucleotide sequences in length [16.9% (0.8 mbsf), 15.2% (5.1 mbsf), 17.2% (18.6 mbsf), 20.2% (48.5 mbsf), and 21.8% (107.0 mbsf)] (Table S1). The genes identified in the metagenomic sequences were then assigned to orthologous groups. The ortholog table and the merged table consisted of 666,560 and 748,988 orthologous groups, respectively. Orthologous groups that contained metagenomic genes were 129,471, among which 47,043 and 82,428 groups were with and without genes from the reference genomes, respectively.

### Frequency of reductive dehalogenase (*rdhA*)-homologous genes

**Figure 1:**
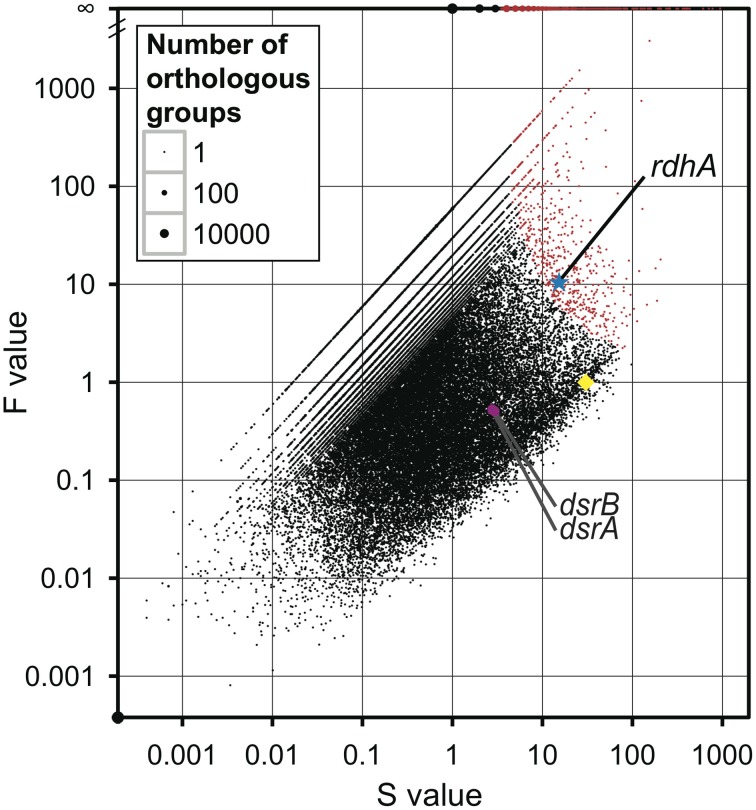
**Comparison of metagenomic samples to previously sequenced genomes for overall orthologous groups.** All groups of genes (orthologous groups) were plotted according to the number of genes assigned to an orthologous group in the metagenomes (S for sum) on the X-axis and the ratio of the frequency of metagenomic genes in an orthologous group to the frequency of genes of reference genomes in the orthologous group (F for fold change) on the Y-axis, with the size of the dot proportional to the number of orthologous groups with the same *S*-and *F*-values. The overrepresented orthologous groups are indicated in red (*p* < 1 × 10^−4^). The star in blue indicates the orthologous group of *rdhA*-homologous genes; the circle in magenta indicates the orthologous group of *dsrA* and *dsrB*; the diamond in yellow indicates the average of the 35 universally conserved single-copy genes.

All of the orthologous groups were plotted using two parameters, *S*-value (“*S*” stands for sum) and *F*-value (“*F*” stands for fold change) (Figure 1). *S*-value is the sum of the average coverage of genes of the five metagenomic pools assigned to an orthologous group, and *F*-value is a fold change of the estimated average copy number of metagenomic genes per genome (average coverage ratio) to the average copy number of genes of reference genomes per genome. The value of average coverage ratio, indicator of the relative copy number of the *rdhA*-homologous genes in the metagenomic samples, is calculated by dividing average coverage of *rdhA* by average of average coverage of the 35 genes (see section Calculation of Average Coverage Ratio). The large *F*-value of a gene indicates the number of the gene found in the metagenomic samples is more frequent than that expected from the distribution of the gene in the present reference genomes. Overrepresented genes in the metagenomic samples were identified using the test for hypergeometric distribution (see section Statistical Analysis), and the overrepresented orthologous groups are indicated in red on the plot (*p* = 10^−4^; false discovery rate-controlled adjusted *p-value* for multiple comparisons). It means the number of false positives under the *p*-value is 1 among 10,000. As 11,167 overrepresented groups were identified in total, only a very few would be false positives.

Interestingly, the orthologous group of *rdhA* genes was one of the highly overrepresented groups in the metagenomic pools (*p* = 1.4 × 10^−07^; Figure 1). This overrepresentation of *rdhA* genes can be ascribed to two factors: the *F*-value (fold change) of *rdhA* is large and the *S*-value (the number of *rdhA* genes) is also considerably large. For comparison, the values of the universally conserved single-copy genes are plotted in Figure 1. The *S*-value of *rdhA* (15.3) was about the half of the average of the *S*-value of the universally conserved single-copy genes (30.4), while the *F*-value of *rdhA* (10.7) was larger than the average of the *F*-value of the universally conserved single-copy genes (by definition, 1). This discrepancy in the *S*- and *F*-values of *rdhA* relative to the universally conserved single-copy genes is ascribed to the fact that the frequency of the *rdhA* genes in the previously sequenced reference genomes was low (less than 0.04 copies per genome in 1806 reference genomes) compared to that in the metagenomic pools (0.5 copies per genome; average coverage ratio defined as the ratio of the *S*-value of *rdhA* to that of the universally conserved single-copy genes). Although the *rdhA* genes were overrepresented among other genes in the metagenomic pools, the average copy number, 0.5 copies per genome, is rather small, considering the fact that it is commonly observed among sequenced genomes that multiple *rdhA* genes are coded in one genome (Richardson, 2013).

**Figure 2:**
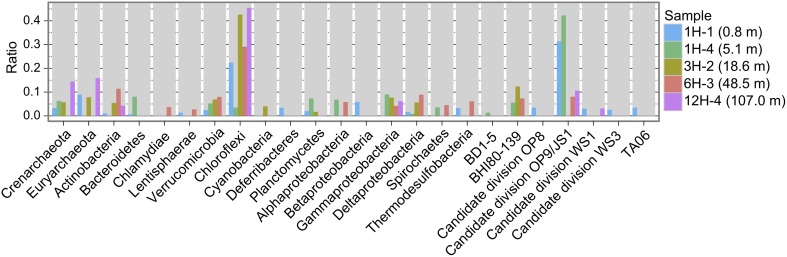
**Taxonomic distribution of 16S rRNA gene fragments.** Plotted is frequency of 16S rRNA gene fragments in the metagenomes to each taxon.

Reductive dehalogenase genes have been reported from relatively limited taxa (Hug et al., 2013). In order to get additional information whether these metagenomic *rdhA*-homologous genes can be derived from known *rdhA*-harboring taxa, 16S rRNA gene fragments detected in the metagenomic pools (Table S1) were classified to taxonomy, allowing us to show the taxonomic distribution pattern (Figure 2). The taxonomic distribution of 16S rRNA gene fragments suggests that about 20–50% of microbial community was derived from phyla that include known *rdhA*-coding organisms (i.e., *Chloroflexi* and *Proteobacteria*) (Figure 2). Organisms of these phyla may code the observed *rdhA*-homologous genes, although we cannot exclude the possibility that organisms of other phyla, from which *rdhA*-coding cultured organisms have not been reported, code the *rdhA*-homologs.

### Distribution of *rdhA*-homologous genes at five sediment depth horizons

**Figure 3:**
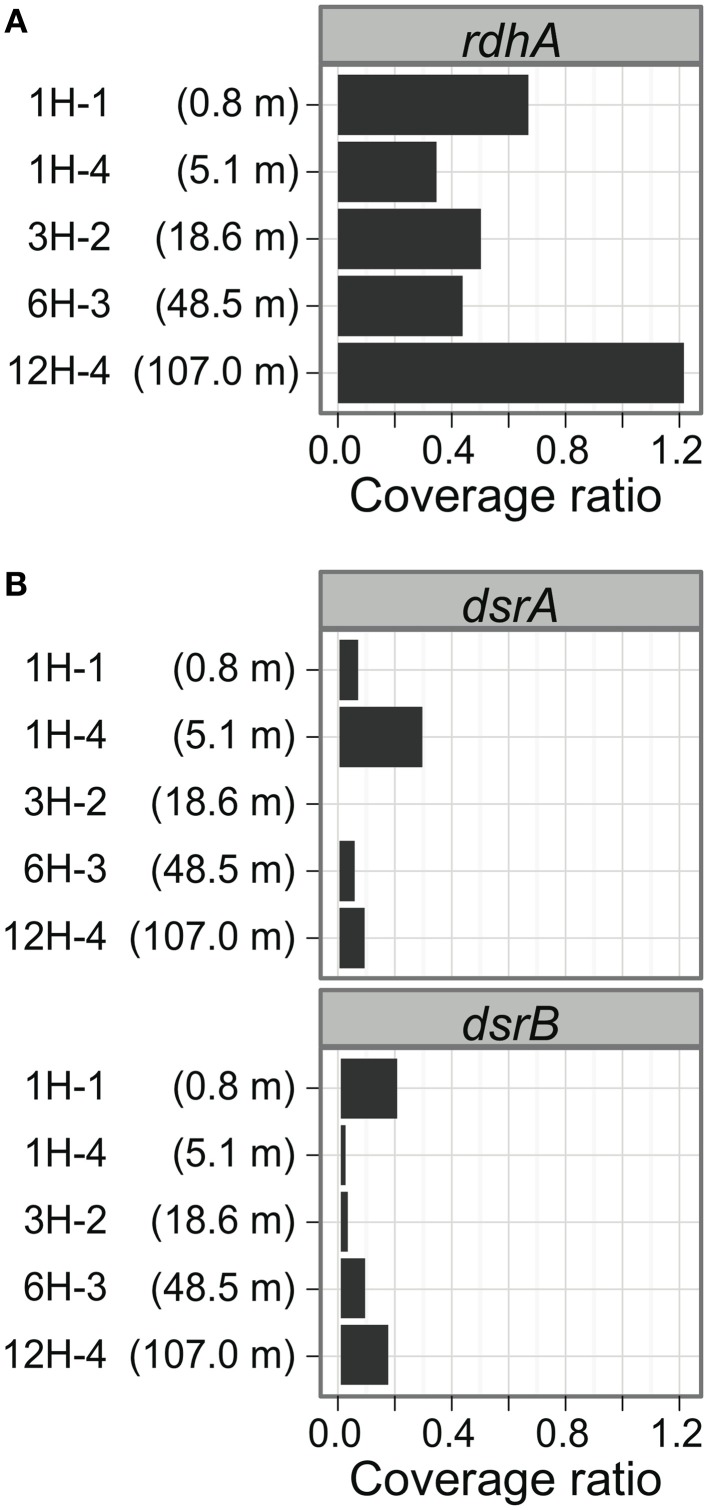
**Distribution of putative *rdhA* and *dsrAB* genes by sediment horizons.** The average coverage ratio, or the ratio of the number of genes relative to universally conserved single-copy genes, is plotted against collection depth. **(A)** *rdhA*-homologous genes. **(B)** *dsrAB* genes.

Examination of the relative copy number of *rdhA*-homologous genes showed that the frequency of *rdhA* homologs is generally similar in metagenomic pools at five sediment depths (Figure 3A). The average coverage ratio indicates the relative copy number of *rdhA*-homologous genes. The value of average coverage ratio is calculated by dividing average coverage of *rdhA* by average of average coverage of the 35 universally conserved single-copy genes, as described in detail in section Calculation of Average Coverage Ratio. In this case, the average coverage of the *rdhA* genes at each horizon was 5.4 (0.8 mbsf), 2.0 (5.1 mbsf), 3.6 (18.6 mbsf), 2.5 (48.5 mbsf), and 4.6 (107.0 mbsf), while the average of the average coverage for the universally conserved single-copy genes was 8.0, 5.7, 7.3, 5.7, 3.8, respectively. Thus, the copy number of *rdhA* genes per genome (average coverage ratio), which is expected to be equivalent to the relative amount of the genes in one cell, was 0.68, 0.35, 0.50, 0.44, 1.2, respectively (Figure 3A).

### Comparison of *dsrAB* distribution to putative *rdhA* distribution

To compare the gene frequency of *rdhA*-homologous genes to other functional genes as the reference, we studied dissimilatory sulfite reductase genes (*dsrAB*), key genes for microbial sulfate reduction. The orthologous groups of *dsrA* and *dsrB* were plotted in Figure 1. The concentration of sulfate in porewater was reported in Tomaru et al. (2009). The amount of *dsrAB* genes indicated by *S*-value is generally small in all samples, which are about one tenth of that of *rdhA*-homologous genes (Figure 3B). It was also found that the frequency of *dsrA* and *dsrB* is not always consistent each other.

### Diversity of *rdhA*-homologous genes

**Figure 4:**
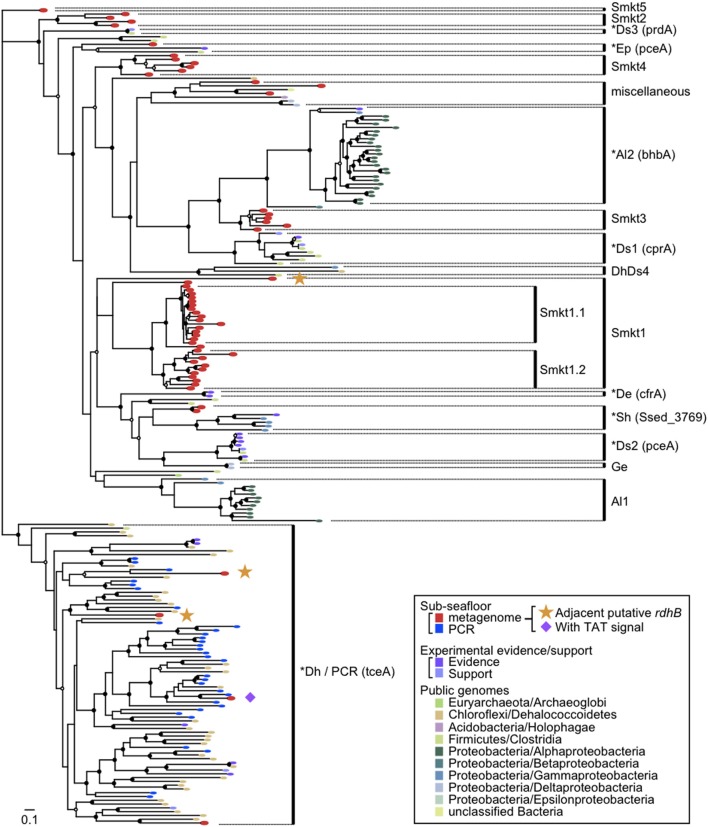
**Diversity of *rdhA*-homologous genes in the sediment samples.** Phylogenetic tree was constructed based on the multiple sequence amino-acid alignment of *rdhA* genes using the FastTree program. Genes included were those detected from the metagenomes of the sediment samples in this study, those detected from the reference genomes, those that are experimentally characterized and are derived from cloned nucleotide sequences, and PCR clones from sediment samples. The previously identified *rdhA* and *rdhA*-homologous genes in the figure are listed in Table S4. PCR clones of the putative *rdhA* genes from sediment samples were described previously (Futagami et al., 2009). These genes were assigned to clusters as indicated in the figure when the support value was ≥0.90, except for Ds3 (0.71), Smkt4 (0.88), miscellaneous (0.15), Smkt1 (0.32), Sh (0.88), Al1 (0.50) to which a cluster name was tentatively assigned. The abbreviation of the cluster names was named after a taxon of which most genes in a cluster belong to, as follows: Smkt, a metagenome from the Shimokita subseafloor sediments in this study; Al, class *Alphaproteobacteria*; De, *Dehalobacter* (phylum *Firmicutes*, class *Clostridia*); Dh, class *Dehalococcoidia* (phylum *Chloroflexi*); Ds, *Desulfitobacterium* (phylum *Firmicutes*, class *Clostridia*); Ep, class *Epsilonproteobacteria*; Ge, *Geobacter* (class *Deltaproteobacteria*); Sh, *Shewanella* (class *Gammaproteobacteria*). A number after the abbreviation denotes the ordinal number of clusters with the abbreviation. A cluster that includes one or more genes with experimental evidence/support is indicated by asterisk at the head and the representative gene name characterized in the parentheses. The references of experimentally characterized RdhA proteins are also listed in Table S4. Taxonomic classifications in the inset indicate phylum names followed by class names. Solid circles and open circles indicate support values of the branch for major splits in the range of 0.90–1.00 and 0.80–0.89, respectively.

A phylogenetic tree was constructed with the *rdhA*-homologous genes in the metagenomes and the public genomes. Sequences obtained from PCR-based molecular ecological surveys as well as several functionally characterized reductive dehalogenase genes were also included in the phylogenetic analysis (Figure 4; the genes used are listed in Table S4).

It has been reported that *rdhA* genes of phylum *Chloroflexi* affiliate to a single large cluster or some distinct small clusters whereas those of phyla *Firmicutes* and *Proteobacteria* are scattered to affiliate within different clusters (Hug et al., 2013).

The tree revealed several clusters, of which some were newly identified, whereas the others correspond to known clusters or a few genes in the previous report. Notably most of the newly identified clusters (labeled Smkt1, Smkt2, Smkt3, and Smkt4 in Figure 4; Smkt, a metagenome from the Shimokita subseafloor sediments in this study; a number after the abbreviation denotes the ordinal number of clusters with the abbreviation) were solely composed of genes from the present metagenomes. These clusters are phylogenetically distinct from the clusters to which previously known genes are classified. On the other hand, the PCR products of *rdhA* genes from the subseafloor sediments all clustered with those from the genera *Dehalococcoides* and *Dehalogenimonas* of the class *Dehalococcoidia* likely due to the bias of PCR primers, which is somewhat consistent with the findings of a previous report (Futagami et al., 2009). On the phylogenetic tree, we identified genes that form a cluster (a subtree), whether they are metagenomic genes or not. For example, a cluster that does not include genes from the present metagenomes [labeled as Al2 in Figure 4; the abbreviation of the cluster name was named after the taxon (class *Alphaproteobacteria*) of which most genes in the cluster belong to; a number after the abbreviation denotes the ordinal number of clusters with the abbreviation] corresponds to that described recently (Chen et al., 2013) where *bhbA* gene is proven to be a reductive dehalogenase for brominated aromatic compounds.

**Figure 5:**
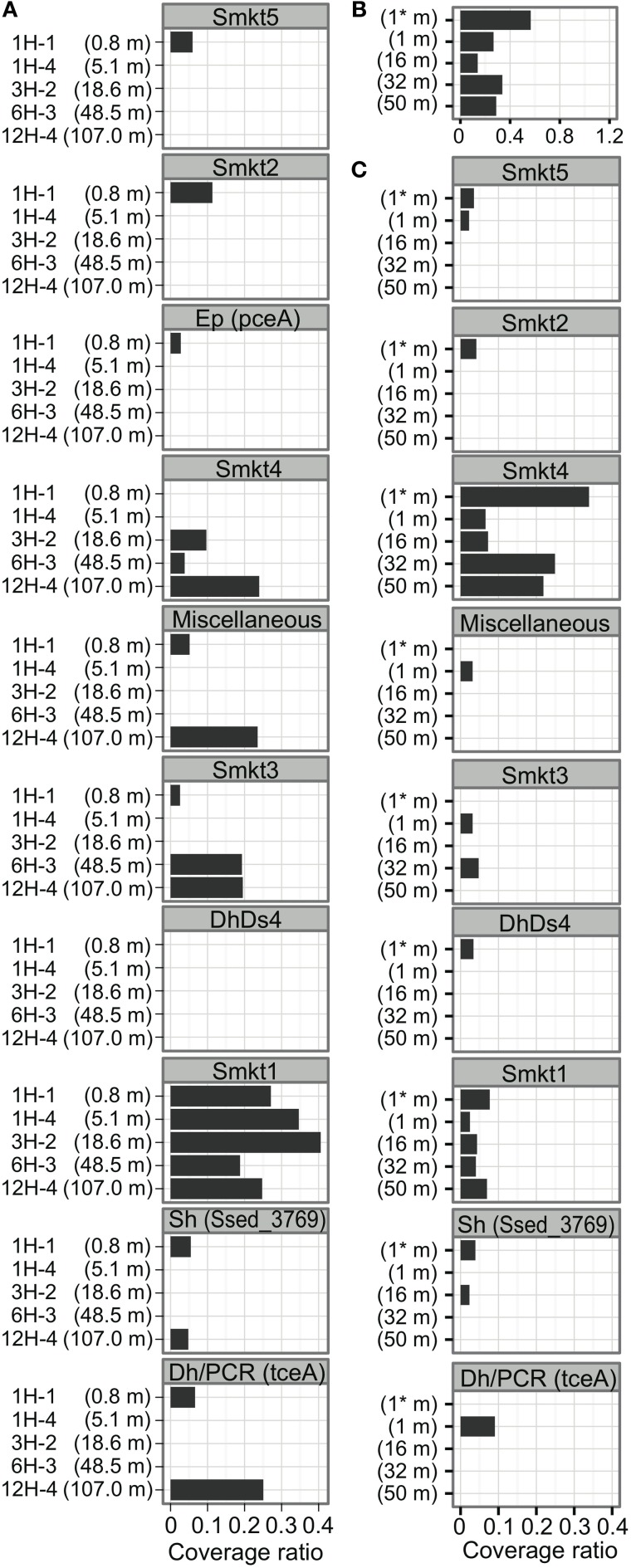
**Distribution of *rdhA*-homologous genes at Site C9001 off Shimokita Peninsula and at Peru-margin. (A)** Distribution of *rdhA*-homologous genes of clusters indicated in Figure 4 among the five horizons from different depths of the Shimokita metagenomes in the present study. The number is the average coverage ratio as in Figure 3. The order of clusters corresponds to that in Figure 4. The abbreviation of cluster names are defined in Figure 4 as follows: Smkt, a metagenome from the Shimokita subseafloor sediments in this study; Al, class *Alphaproteobacteria*; Dh, class *Dehalococcoidia* (phylum *Chloroflexi*); Ds, *Desulfitobacterium* (phylum *Firmicutes*, class *Clostridia*); Ep, class *Epsilonproteobacteria*; Sh, *Shewanella* (class *Gammaproteobacteria*). A number after the abbreviation denotes the ordinal number of clusters with the abbreviation. **(B)** Distribution of *rdhA*-homologous genes in the Peru-margin subseafloor metagenomes. Asterisk (*) denotes original, unamplified sample (Biddle et al., 2008). **(C)** Distribution of *rdhA*-homologous genes of clusters in the Peru-margin subseafloor metagenomes.

It is clear from the phylogenetic tree that the number of *rdhA* genes within the new metagenomic cluster Smkt1 is larger than the number classified to the Dh/PCR cluster (only four genes for the latter were detected from the metagenomes (Figure 4); Dh, the class *Dehalococcoidia* (within the phylum *Chloroflexi*). The frequency of genes of the cluster Smkt1 was more or less similar at all five depths examined (Figure 5A). Other clusters were generally composed of fewer genes.

The *rdhA* genes in the same clusters were derived from both shallow and deep sediments (Smkt1, Smkt4, Dh/PCR, Sh, miscellaneous; Figure 5A; Sh, *Shewanella* the class *Gammaproteobacteria*; Ep, the class *Epsilonproteobacteria*). Even subclusters Smkt1.1 and Smkt1.2 included genes from both shallow and deep sediments (Figure 4).

### Protein domain organization of *rdhA*-homologous genes

**Figure 6:**
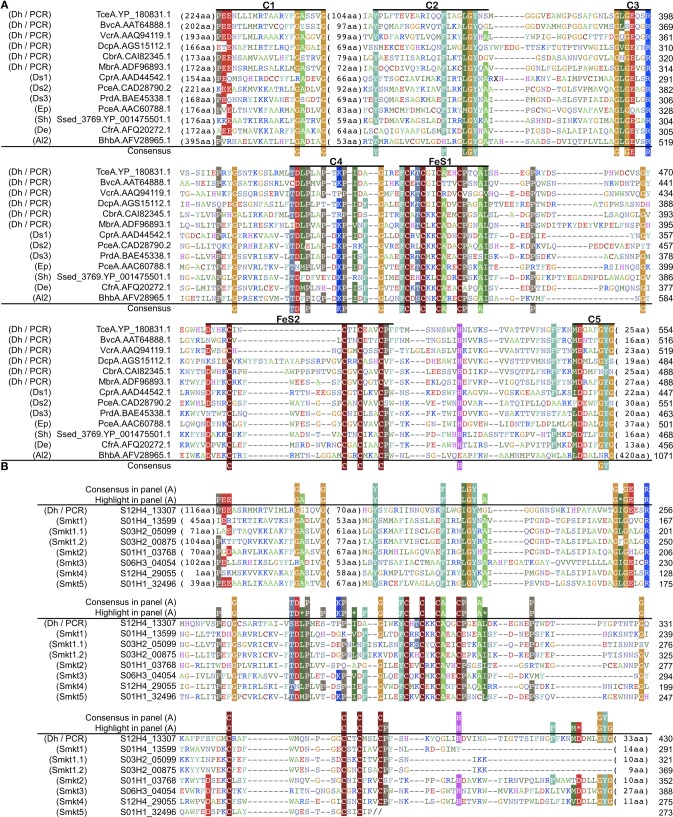
**Conserved regions of amino acids sequences of *rdhA* genes. (A)** Regions conserved among experimentally characterized reductive dehalogenases. The members are indicated by their name and protein accession. A label in the parentheses indicates the cluster name as in Figures 4, 5. Amino acid residues are colored according to the similarity of their physico-chemical properties. Conserved regions and two Fe-S binding motifs are labeled as C1 through C5 and FeS (FeS1 and FeS2), respectively. Consensus residues are those conserved in ≥80% (10 or more among 13 enzymes); highlighted residues are those conserved in ≥60% (7 or more among 13). Experimental evidences have been reported for the following proteins: TceA.YP_180831.1 (Magnuson et al., 1998), VcrA.AAQ94119.1 (Müller et al., 2004), DcpA.AGS15112.1, CbrA.CAI82345.1 (Padilla-Crespo et al., 2014), CprA.AAD44542.1 (van de Pas et al., 1999; Smidt et al., 2000), PceA.CAD28790.2 (Maillard et al., 2003), PceA.AAC60788.1 (Neumann et al., 1998), Ssed_3769.YP_001475501.1 (Lohner and Spormann, 2013), CfrA.AFQ20272.1 (Tang and Edwards, 2013), BhbA.AFV28965.1 (Chen et al., 2013). Experimental support (transcription under organohalide compounds) has been reported for the following proteins: BvcA.AAT64888.1 (Krajmalnik-Brown et al., 2004). MbrA.ADF96893.1 (Chow et al., 2010), PrdA.BAE45338.1 (Tsukagoshi et al., 2006). **(B)** Residues of the *rdhA*-homologous genes in the sediment samples around the conserved regions defined in **(A)**. The members are indicated by locus tag. A label in the parentheses indicates the cluster name as in Figures 4, 5. Double slash characters “//” denotes truncation at the end of the nucleotide sequence.

Amino acid sequence motifs in the predicted products for *rdhA* genes were examined to further characterize metagenome-specific *rdhA* gene products (sequences within clusters Smkt1, Smkt2, Smkt3, Smkt4, and Smkt5). Experimentally characterized typical reductive dehalogenase sequence has an RdhA domain that consists of 2 × Fe-S binding motif and other conserved regions at the middle of the gene product [Figure 6A; denoted as FeS (FeS1 and FeS2) and C1 through C5, respectively] (Smidt and de Vos, 2004; Hug et al., 2013). Most RdhA sequences known so far also encode twin arginine motif (TAT motif) at the N-terminus (Smidt and de Vos, 2004). The lack of TAT signal in the *rdhA* homolog is reported in *Anaeromyxobacter dehalogenans* 2CP-C (Thomas et al., 2008) and *Dehalogenimonas lykanthroporepellens* BL-DC-9 (Siddaramappa et al., 2012).

The Rdh domain was detected in metagenomic sequences of the present study (Table 1 and Figure 6B). Examination of multiple alignment revealed that two Fe-S cluster-binding motifs were found in those RdhA sequences as well as all the other conserved regions but C5 region in the clusters Smkt1 and Smkt5 (Figure 6B). Interestingly, however, genes of the cluster Smkt1 lacked twin arginine motif (TAT motif) (Table 1). Lack of a TAT signal sequence was confirmed by the N-terminal alignment of the gene products in the cluster Smkt1 to those from other clusters. As RdhA proteins are transported across the cytoplasmic membrane by action of the Tat transport system recognizing the TAT motif (Palmer and Berks, 2003), whether those *rdhA*-homologous genes lacking an apparent TAT motif are targeted to the membrane remains a further question. It does not necessarily mean that the product of those *rdhA*-homologous genes are not targeted to the membrane by the Tat transport system, because it is known that there are cases in which the physiological mechanism for targeting a protein to the Tat system is less obvious (Berks et al., 2005).

**Table 1:** **Status of *rdhA*–homologous genes in the metagenome**.

**Cluster**	**Number of genes**			
	**Rdh motif^a^**	**2 × Fe-S motif^b^**	**TAT signal^c^**	**Adjacent *rdhB***
Typical RdhA	+	+	+	+
Smkt1	9/12	12/12	0/12	1
Smkt2	2/2	2/2	0/1	
Smkt3	2/2	2/2	0/1	
Smkt4	5/5	5/5	N.A.	
Smkt5	1/1	1/1	N.A.	
Dh/PCR	2/2	2/2	1/1	2
Ep	1/1	1/1	N.A.	
Sh	N.A.	1/1	N.A.	
Miscellaneous	2/2	2/2	N.A.	

Number of genes with the motif/number of genes of which corresponding region was sequenced.

a Number of genes that had a motif RDH (TIGR02486).

b Number of genes that had conserved two sets of four cysteine residues (two Fe-S motifs).

c Number of genes that had a TAT signal motif (TIGR01409 or PF10518.3). Only those with start codon were examined.

N.A., not applicable.

### The *rdhB* homologs, anchoring protein-coding gene, adjacent to *rdhA*-homologous gene

Most of the experimentally characterized *rdhA* genes are closely linked with a small open reading frame (designated *rdhB*) that codes for a hydrophobic membrane protein, typically about 100-amino acid hydrophobic protein (Smidt and de Vos, 2004), which is predicted to have three transmembrane α-helices (Maillard et al., 2003). It is conceivable that these helices anchor RdhA to the cytoplasmic membrane (Smidt and de Vos, 2004). Co-transcription of both genes has been shown for some cases such as *pceAB* (Neumann et al., 1998) and *cprBA* (Smidt et al., 2000), indicating a functional link of both gene products, although experimental characterization of association of these proteins is yet to be shown.

The examination of *rdhB*-homologous genes in the reference genomes of these experimentally supported *rdhB* homologs revealed that sequence similarity among them was mostly below the threshold used to construct the orthlog table in this study and they formed separate orthologous groups that consist of a single gene.

As an approach based only on sequence similarity turned out to be prone to miss *rdhB*–homologous genes, another approach based on gene-neighborhood was conducted to find *rdhB* homologs by introducing the following criteria: (1) genes within 2 kb of the *rdhA*-homologous genes, (2) genes with predicted amino acid sequences shorter than 150 amino acids, and (3) genes with predicted amino acid sequences of two to three transmembrane regions. The survey revealed that three *rdhA*-homologous genes in the metagenomes in this study had a gene that satisfied the above criteria, so was regarded as the *rdhB*-homologous genes (Tables 1, S5). We note that homologs of the genes that satisfied the criteria in the reference genomes were all flanked with a putative *rdhA*.

One of these three *rdhB*-homologous genes was adjacent to an *rdhA* of the cluster Smkt1 (Figure 4). The presence of *rdhB* homolog suggests that the function of the Smkt1 gene is similar to other *rdhA*-homologous genes, although frequency of the *rdhB* was apparently low as compared to that of the Smkt1 sequence. The other two *rdhB*-homologous genes from the metagenomes were adjacent to the *rdhA* gene of the cluster Dh/PCR. This result agrees well with the fact that *rdhA* genes from the reference genomes in this cluster are mostly adjacent to putative *rdhB*.

Other neighborhood genes, which are identified on the nucleotide sequences that code the metagenomic *rdhA* genes, did not code any genes related to mobile elements (transposases or phages) including integrases. It is worth noting that only very limited portion of the flanking region of the metagenomic *rdhA* homologs were sequenced (N50 was only 1.5–1.7 kb).

### Comparison of *rdhA* distribution between off shimokita and peru

To compare the gene frequency of *rdhA*-homologous genes to geologically distinct location, we analyzed frequency of the *rdhA* homologs in metagenomic sequences from Peru margin (Biddle et al., 2008). It was analyzed similarly as above, although the Peru margin metagenomic sequence pools consist of relatively short sequence reads for the accurate assignment of orthologous groups. The analysis revealed that the frequency of *rdhA*-homologous genes in the Peru margin sediments was generally similar to that in the present Shimokita sediments (Figures 3, 5B). Distribution among different depths was rather even, as that in the present Shimokita sediments. Nevertheless, phylogenetic composition of the genes in Peruvian sediments was found to be slightly different from Shimokita, i.e., genes assigned to Smkt1 were fewer whereas those assigned to Smkt4 were higher (Figures 5A,C).

## Discussion

This study clearly demonstrated that the PCR-independent metagenomic approach is a powerful tool for characterizing diversity and distribution pattern of the target gene(s) from microbial communities in the deep subseafloor sedimentary biosphere (see, Biddle et al., 2008, 2011). Through the metagenomic approach, the wide range of previously undiscovered *rdhA*-like sequences could successfully be recovered, suggesting that reductive dehalogenase-related pathways were more genetically diverse than previously expected. It has also been evidenced that metagenomic pools from marine subsurface sediments contain novel and hence previously unidentified/uncharacterized *rdhA*-homologous genes, which were found to be more diverse than those detected by PCR-based molecular ecological survey (Futagami et al., 2009). The lack of a TAT motif in the sequences in new clusters can reasonably explain that no amplification of the new cluster genes occurs by PCR using a primer (RRF2) that covers the conserved TAT motif region (Krajmalnik-Brown et al., 2004).

The combined use of an ortholog table and statistical evaluation enabled quantitative assay of the target gene(s) (as an orthologous group) in metagenomic pools, revealing that *rdhA*-homologs are overrepresented among other genes. Further, the *rdhA*-homologous genes showed phylogenetically diverse and spatially distribute at five different depths from near the seafloor to a depth of 107.0 mbsf, at least 150,000-year-old sediment (Domitsu et al., 2010). The *rdhA*-homologous genes of the cluster Smkt1.1 and Smkt1.2 were consistently detected throughout the depths examined. The widespread occurrence of microorganisms that code *rdhA*-homologous genes in the Pacific margins is further supported by the comparison of the metagenomic pools of the sediments off Shimokita and off Peru. Taken together, our data indicate that the heterotrophic metabolic processes mediated by the *rdhA*-homologous genes can be ecologically important, functioning in the organic-rich subseafloor biosphere.

As anaerobic organohalide-respiring bacteria utilize organohalides as the electron acceptor through the enzymatic reaction of Rdh proteins (Smidt and de Vos, 2004), the high frequency of *rdhA*-homologous genes with relatively constant distribution in the sediment column suggests that there are still bio-available organohalides to sustain the organohalide respiring prokaryotes. The relatively constant value of total organic carbon at depths surrounding the 5 depths, around 1–2% [e.g., 2.3% (0.2 mbsf), 1.2% (18.0 mbsf), 1.3% (37.2 mbsf), 1.8% (56.3 mbsf), and 1.9% (113.2 mbsf)] (Aoike, 2007), does not contradict to this possibility. It is worth noting that the depositional environment thousands of years ago must predate the accumulation of anthropogenic organohalides; therefore, the subseafloor *rdhA*-homologs are not associated with polluted terrestrial environments. Possible sources of structurally diverse organohalides such as halogenated aromatic compounds and halogenated alkanes can be a benthic biological origin, such as hemichordates and marine sponges (King, 1986; Harper, 2000; Winterton, 2000; Öberg, 2002). One of other possible sources of naturally derived organohalides is the chlorinated degradation products of lignin, the most abundant aromatic substance on our planet (Boerjan et al., 2003). A not yet elucidated degradation process that is similar to lignin degradation could be taking place in seawater or in benthic depositional systems (i.e., transfer of chlorine from inorganic to organic forms). This could be similar to the process that produces massive amounts of organic carbon that are mineralized to refractory forms during depositional processes (Jiao et al., 2010). The *rdhA*-homologous genes were also detected in the open Pacific Ocean, far away from the continental margins (Futagami et al., 2009), suggesting the possibility of not yet elucidated mechanisms for the formation of organohalides in the seawater-sediment system.

In addition, we compared frequencies of *rdhA* homologs to *dsrAB* among different depths. Interestingly, the result showed that frequency of *rdhA*-homologous genes is generally much higher than that of *dsrAB* (Figures 3A,B). We observed either or both *dsrA* and *dsrB* genes in all the samples examined, which phenomenon is in good agreement with previous reports regarding the widespread occurrence of the genes, even in sulfate-depleted deep horizons (Schippers and Neretin, 2006). We have to note here that organohalide-respiring bacteria such as the members of *Dehalococcoides* code multiple copies of the *rdhA*-homologs while most of sulfate reducing bacteria do a single copy of the *dsrAB* genes. This indicates that remarkably high frequency of diverse *rdhA*-homologous does not necessarily correspond to the cell abundance of organohalide-respiring microbes.

## Conclusions and research perspectives

Through metagenomic analysis of the deep sedimentary habitats, we found that remarkably diverse putative *rdhA* genes are spatially widespread in shallow to deep and old sedimentary habitats on the Pacific margins. We identified four new phylogenetic clusters of putative *rdhA* genes that are solely composed of genes detectable only from PCR-independent metagenomic analysis. These findings suggest that the deeply buried organohalide compounds may play ecologically significant roles in the subseafloor biogeochemical carbon cycle, and the anaerobic organohalide respiration is potentially one of the key metabolic energy-yielding mechanisms of the organic compound-fueled deep subseafloor microbial ecosystem.

However, the ecological consequences of organohalide degradation in the deep subseafloor biosphere still remain largely unknown: why are such phylogenetically diverse *rdhA*-like genes present? What kinds of organohalides exist and in what amounts? To what depths and at what levels do such organohalide-utilizing microbes occur? If all bio-available organohalide compounds are consumed, do these microbes disappear? What are the *in situ* dehalogenation activities and the impacts of biogeochemical halogen cycles? What are the possible electron donors for subseafloor organohalide respiration? These questions need to be addressed through advanced microbiological and biogeochemical analyses in the future. In addition, metabolic functioning of diverse *rdhA*-homologous genes as well as the gene expression and activity *in situ* still remains largely unknown, which should be clarified by culture-dependent and -independent molecular ecological studies at single cells to community levels. In addition, metagenomic analysis of deep subseafloor microbial communities is one of the technological challenges. In this study, except for a near surface sample, we performed multiple MDA reactions for the preparation of metagenomic libraries to reduce possible bias randomly occurred by MDA, which may still remain uncertainty for the accurate estimate and even coverage of the environmental gene pool. This means that development of molecular ecological technologies, such as high-sensitivity, high-throughput, less-bias molecular tools, should be necessary for the future deep-biosphere exploration.

## Conflict of interest statement

The authors declare that the research was conducted in the absence of any commercial or financial relationships that could be construed as a potential conflict of interest.

## Supplementary material

Table S1 **Summary of sequencing characteristics**.

Table S2 **List of genomic sequences used for the construction of the ortholog table**.

Table S3 **Universally conserved single-copy genes in the ortholog table**.

Table S4 **List of *rdhA* and its homologs used in Figure 4**.

Table S5 **List of putative *rdhB* gene sequences**.
